# Quality of Life in Nursing Professionals: Burnout, Fatigue, and Compassion Satisfaction

**DOI:** 10.3390/ijerph17041253

**Published:** 2020-02-15

**Authors:** María Dolores Ruiz-Fernández, Esteban Pérez-García, Ángela María Ortega-Galán

**Affiliations:** 1 Department of Nursing Science, Physiotherapy and Medicine, University of Almeria, 04120 Almeria, Spain; 2 Hospital Infanta Elena, Andalusian Health Service, 21080 Huelva, Spain; 3 Department of Nursing, University of Huelva, 21071 Huelva, Spain; angela.ortega@denf.uhu.es

**Keywords:** nursing, compassion fatigue, compassion satisfaction, burnout, socio-demographic factors, work-related factors

## Abstract

The objective of this research was to analyse the quality of life of nursing professionals and its relationship with socio-demographic variables and the work context. A multi-centre, descriptive, cross-sectional design was used. Questionnaires were administered to 1521 nurses working in the Andalusian Public Health System (APHS), Spain. Professional quality of life (ProQOL v. IV) was measured, as well as several socio-demographic and work-related variables. To this end, a descriptive analysis and multiple exploratory analyses were conducted. The levels of compassion fatigue (CF) and burnout (BO) were elevated. The level of compassion satisfaction (CS) was below the estimated mean. Marital status, the healthcare setting, the area where the centre is located, and the work shift are variables associated with CF. According to the multiple linear regression model, the predicting factors for the occurrence of CF were being married, working in primary care, in urban areas, and working a morning/evening/night shift. The variables related to CS were the professional’s age, sex, marital status, the healthcare setting of the centre, the location of the centre, and the work shift. Specifically, according to the exploratory model, the factors that predicted a reduction in CS were working in primary care, in urban areas, and working a morning/evening/night shift. However, being divorced increased CS. BO was influenced only by the work shift. Nursing professionals are exposed to certain factors that may influence professional quality of life. Some of these factors are related to the work context.

## 1. Introduction

Healthcare professionals are exposed to several elements derived from work-related circumstances that contribute to a complex work environment [1]. These elements may include, but are not limited to, excessive workload, shift rotation, and work setting [2,3]. Healthcare professionals, especially nurses, witness the pain and suffering of their patients in the daily practice of their work [4,5]. Providing care in these cases is very demanding for the healthcare community in physical, emotional, and spiritual terms [6]. Their work requires certain qualities, such as empathy, compassion, and closeness to the individuals and families they care for [7].

In the current healthcare context, signs of occupational stress and burnout (BO) have been reported and have even been described as public health problems [8]. Simply observing the reality in hospital care and primary care settings suffices to confirm that health professionals are prone to experience situations of stress or fatigue with negative consequences [9,10,11]. Repeated exposure to unpredictable challenges in nursing practice may cause symptoms of anxiety, exhaustion, and stress in professionals [12]. At the same time, job satisfaction and the quality of the care provided decrease [13]. The negative consequences of the work context may cause workers to feel exhausted and to suffer from certain syndromes, such as BO [14,15].

The concept of BO, also known as “burnout syndrome”, was first described by Freudenberger (1974) [16] as a feeling of failure and exhaustion resulting from excessive demands on the energy, personal resources or spiritual strength of workers that prevents them from providing care and assistance to users of organisations in different fields whose primary objective is to help others. The first lines of research on this syndrome focused on healthcare-related occupations that provided services to individuals in need for care. These lines of research subsequently spread to other occupational sectors [17]. There is no agreement in the literature on a single definition of BO syndrome. However, the most widely accepted approach to this term conceptualises it as a multidimensional process with three main constructs: emotional fatigue or exhaustion, depersonalisation, and personal accomplishment [18,19]. According to this conceptual model, BO has recently been defined as a syndrome of emotional exhaustion, depersonalisation, and lack of personal fulfilment at work, with these characteristics developing as a result of continued exposure to work stressors [14].

Another syndrome related to BO is compassion fatigue (CF). This concept has gained considerable attention over the past twenty years, as health professionals have increasingly been challenged to manage complex demands in an overburdened healthcare system [20]. CF is defined by Figley [15] as the cost of caring for others or for their emotional pain resulting from the desire to help individuals suffering traumatic events. Other synonyms include “empathy burnout”, “vicarious traumatisation”, or “secondary traumatic stress”, all of which creates some degree of conceptual confusion. However, CF is a concept of documented relevance to those in nursing and represents a basic inability to nurture others and engenders a temporal component [20]. The literature related to CF essentially adheres to “the phenomenon as a state of exhaustion that is dependent on a caring relationship” with a loss of coping ability to continued exposure to patient suffering and death [21]. Thus, professionals suffering from CF may experience feelings of fear or dread when interacting with the patients they care for, which may lead to avoidance behaviours in the professional-patient relationship [22,23].

A protective factor against CF is compassion satisfaction (CS), described as the satisfaction experienced by healthcare professionals when performing their work properly, which also includes satisfaction with their relationship with colleagues and the sense that the work they perform is of social value [24]. This construct focuses on the satisfaction that comes from helping and caring for individuals in difficult situations [25]. Unsurprisingly, the balance between CS and CF determines the level of professional quality of life [26].

Certain work-related factors may influence quality of life among professionals. Specifically, CF and CS have been reported to be related to the healthcare setting and the work environment in certain healthcare services [27]. A study involving oncology nurses found years of work experience and working in secondary hospitals to be factors that influence the occurrence of CF [28]. Age, sex, marital status, seniority, years of experience, and shifts are variables that may be related to CF, CS, and BO in paediatric and critical care nurses [24,26,29]. However, studies so far have been conducted with nurses working in highly specific services, and not at a general level [24,26,29,30,31]. In addition, previous studies analysing these factors do not determine exactly what socio-demographic and work-related variables may influence these syndromes, which are related to the work context to which nursing professionals are exposed [13,32]. Workplace violence is also a widespread risk factor for healthcare professionals, which is related to stress, well-being, and quality of life [33,34]. For these reasons, this study set out to analyse the quality of life of professionals and its links to socio-demographic variables and work context among nursing professionals working in different care settings in the Andalusian Public Health System (Spain).

## 2. Materials and Methods

### 2.1. Design

A multi-centre, descriptive, cross-sectional design was used.

### 2.2. Participants

The study sample consisted of 1521 nurses working in the Andalusian Public Health System (APHS), Spain, both in the hospital and in the primary healthcare settings. The sample size necessary was calculated based on the number of nurses who worked at the APHS in 2015 (N = 22,533) with a two-tailed 95% confidence interval, with an accuracy level of 3%, assuming that the expected BO rate was 32.20% [35]. The inclusion criterion was being an active nursing professional currently working in healthcare services that facilitated direct contact with patients. Professionals working in healthcare administration management or in very specific services where there was no direct contact with patients (laboratory services, sterilisation, etc.) were excluded.

### 2.3. Instruments

A socio-demographic and work-related data collection sheet was prepared, including the following: age, sex (female, male), marital status (married, single, divorced, other), employment status (casual, temporary or long-term, statutory or permanent), healthcare setting (primary care, hospital care), area where the healthcare centre is located (urban area or area with more than 10,000 inhabitants; semi-urban area, between 5000 and 10,000 inhabitants; rural area or area with fewer than 5000 inhabitants), work shift (mornings, evenings, mornings/evenings, mornings/evenings/nights, 12-h, nights, other), work experience (months), and seniority in the current position (months).

The Professional Quality of Life Scale (ProQOL v. IV) [36] is used in health and social care professionals who are exposed to situations of trauma and suffering. This questionnaire was translated into Spanish by Morante Moreno and Rodríguez [37] and has been used in healthcare professionals [38]. This is a self-administered questionnaire consisting of 30 items rated on a 5-point Likert scale (ranging from 1 = “never” to 5 = “very often”). The scale is divided into three subscales: Compassion fatigue (10 items), Compassion satisfaction (10 items), and Burnout (10 items). Higher scores on each subscale indicate higher CF, CS, and BO values. The mean score is 13 for the CF subscale, 37 for the CS subscale, and 22 for the BO subscale. In Stamm [26], Cronbach’s alpha values were reported of 0.80 for CF, 0.89 for CS, and 0.71 for BO, respectively.

### 2.4. Procedure

Data were collected from January to December 2018. Researchers from the 8 provinces of the Autonomous Community of Andalusia, Spain, collaborated in the study. The questionnaires were administered in the different healthcare centres in the APHS: in total, 12 hospitals, 26 primary care districts, and 10 healthcare management areas (including primary care and hospital care). To this end, the researchers were briefed on the administration of the questionnaires. The estimated completion time was 15 min. Participation was voluntary and anonymous. Participants were informed about the purpose of the study and were requested to give their informed consent in writing. Approval was obtained from the Research Ethics Committee of Almería Centro (CEI-27 September 2017), which was extended to the rest of Andalusia as a single authorisation. The ethical principles enshrined in the Declaration of Helsinki were observed at all times. The study complied with the national personal data protection regulations in force (Spanish Organic Law 3/2018 on Personal Data Protection and Guarantee of Digital Rights).

### 2.5. Data Analysis

The means and standard deviations of the quantitative variables, as well as the absolute values and percentages of the qualitative variables, were analysed. Fulfilment of the normality criterion was checked using the Kolmogorov-Smirnov test. Although some of the variables did not comply with this criterion, parametric tests were performed in accordance with the central limit theorem [39]. Both parametric and nonparametric tests were convergent. For the comparison of means, Student’s *t*-test for independent samples and one-way ANOVA were used. In each case a threshold of 95% was used to determine statistical significance. For the analysis of bivariate correlations, Pearson’s correlation was used. Subsequently, the multiple comparisons or post-hoc Significant Mean Differences (SMD) test was used in order to determine exactly in which groups the SMDs were present. Finally, a stepwise linear regression model was created using the least squares method. The dependent variables were the different sub-dimensions of professional quality of life (ProQOL) (CF, CS, and BO), and the explanatory variables were the most significant variables of the bivariate analysis [40]. Goodness of fit was determined using the coefficient of determination (R2) and its adjusted version. The statistical program SPSS Statistics v.25 (IBM Corp., Armonk, NY, USA) was used for data processing.

## 3. Results

### 3.1. Characteristics of the Participants

The mean age of the participants was 47.32 (SD = 8.44) years of age, ranging from 23 to 64 years of age. The majority of the participants were women (75.5%), married (69.8%), and had a stable employment status (59.5%). The majority of professionals worked in hospital settings (55%) and the majority of the centres were located in urban areas (89.5%). Their most frequent work shifts were the morning shift (32.1%) and the morning/evening/night shift (29%). Their mean work experience was 275.36 months (SD = 110.42) and their mean seniority in the current job was 146.81 months (SD = 117.52) (Table 1).

### 3.2. Descriptive and Bivariate Analyses of Socio-Demographic and Work-Related Variables and Quality of Life Sub-Dimensions

The mean score for CF was 20.74 (SD = 7.88), the mean score for CS was 35.48 (SD = 7.39), and the mean score for BO was 23.44 (SD = 5.29). Table 2 shows that there are significant differences in the mean scores for CF for marital status (F = 3.33; *p* = 0.01). Specifically, according to the post-hoc DMS tests, the mean score for CF is significantly higher in married participants than in divorced participants (DMS = 2.32; *p* ≤ 0.05). Regarding the healthcare setting, the mean score for CF is significantly higher in primary care than in hospital care (t = −2.03; *p* = 0.04). With respect to the areas where the healthcare centres are located, differences are observed in the mean scores for CF (F = 3.31; *p* = 0.03). Specifically, according to the post-hoc DMS tests, the mean score for CF is significantly higher in urban areas than in rural areas (DMS = −1.81; *p* ≤ 0.05). In addition, differences in the mean scores for CF between different work shifts are significant (F = 3.45; *p* ≤ 0.05). The post-hoc test indicates that the mean score for CF for CF for the morning/evening shift is significantly higher than for the morning shift (DMS = −1.50; *p* ≤ 0.05), the 12-h shift (DMS = −2.03; *p* ≤ 0.05), and other shifts (DMS = 4.4; *p* ≤ 0.05). In turn, the mean score for CF is significantly higher for the morning/evening/night shift than for the morning shift (DMS = 1.25; *p* ≤ 0.05) and the 12-h shift (DMS = 1.78; *p* ≤ 0.05).

As for the CS variable, there is a significant negative correlation between CS and age: older ages are associated with lower levels of CS (r = −0.61; *p* = 0.01). Differences in mean scores are significant for the sex variable (t = −0.01; *p* = 0.04). Women have significantly higher mean scores than men. There are statistically significant differences regarding marital status (F = 2.95; *p* = 0.03). According to the post-hoc DMS tests, the mean score for CS in divorced participants is significantly higher than in single participants (DMS = 2.50; *p* ≤ 0.05) and in married participants (DMS = 2.12; *p* ≤ 0.05). With respect to the areas where the healthcare centres are located, differences in mean scores are significant (F = 11.83; *p* ≤ 0.01). Specifically, according to the post-hoc DMS tests, these differences are significantly lower in urban areas than in semi-urban areas (DMS = −2.80; *p* ≤ 0.05) and rural areas (DMS = −3.16; *p* ≤ 0.001). In addition, regarding work shifts, differences in mean scores for CS are statistically significant (F = 2.52; *p* = 0.01). The mean score for the morning shift is significantly higher than the mean score for the morning/evening shift (DMS = 1.53; *p* ≤ 0.05) and for the morning/evening/night shift (DMS = 1.44; *p* ≤ 0.05). The mean score for the 12-h shift was significantly higher than the mean score for the morning/evening shift (DMS = 1.48; *p* ≤ 0.05) and the morning/evening/night shift (DMS = 1.39; *p* ≤ 0.05).

As for the BO sub-dimension, differences in mean scores are significant depending on the work shift (F = 2.44; *p* = 0.02). Specifically, the mean scores for BO are lower for the morning shift than for the morning/evening/night shift (DMS = −1.04; *p* ≤ 0.05).

### 3.3. Exploratory Model with Socio-Demographic and Work-Related Variables, and Quality of Life Sub-Dimensions

In the linear regression, four models were generated, with model 4 having the greatest predictive capacity of CF variability (adjusted R^2^ = 0.02; *p* ≤ 0.05) (Table 3). The significant predictive variables of this model were the following, in order of importance: primary care (B = 0.11; *p* ≤ 0.001), urban location (B = 0.09; *p* ≤ 0.001), morning/evening/night shift (B = 0.08; *p* ≤ 0.001), and being married (B = 0.06; *p* ≤ 0.001). The ANOVA test indicates that there is a significant linear relationship between the dependent variable and the set of explanatory variables of this model (F = 8.04; *p* ≤ 0.001).

With respect to the CS sub-dimension, 5 models were generated, with model 5 having the greatest predictive capacity (adjusted R^2^ = 0.04; *p* ≤ 0.05) (Table 4). The significant variables that explain the model negatively, i.e., each of these variables decreases the level of CS irrespective of the rest of the variables, are as follows: urban area (B = −0.14; *p* ≤ 0.001), primary care, (B = −0.11; *p* ≤ 0.001), morning/evening/night shift (B = −0.08; *p* ≤ 0.001), and age (B = −0.06; *p* ≤ 0.001). On the other hand, being divorced increases the level of CS irrespective of the rest of the variables (B = 0.07; *p* ≤ 0.001). According to the ANOVA test, the linear relationship is significant between the dependent variable and the set of explanatory variables of this model (F = 11.38; *p* ≤ 0.001).

According to Table 5, 4% of this model would explain the variance of the dependent variable, BO (adjusted R^2^ = 0.04; *p* ≤ 0.05). The morning/evening/night shift would be a predictive factor of this model (B = 0.66.; *p* ≤ 0.05).

## 4. Discussion

The results of this research determine the existence of CF and BO among nursing professionals [35,36], as well as mean CS values lower than the reference values [41]. In addition, certain socio-demographic and work-related characteristics determine the presence of CF and influence CS and, to a lesser extent, BO. We believe that these professionals may benefit from interventions aimed at enhancing their compassion, self-compassion, and empathy skills. In fact, recent studies on the effects of mindfulness and compassion programmes show that such programmes have led to a decrease in symptoms of depression, depersonalisation, and emotional burnout, and to an increase in emotion regulation skills, higher levels of self-care, and enhanced communication skills at work [42,43,44].

Age is a variable that has not been shown to be positively correlated with CF in this study. These data are in line with previous research [28,45]. However, other studies found that age was a predictor of CF. The relationship between age and CF therefore remains unclear. Certain authors have indicated that age and CF were positively correlated [46,47], with older ages indicating higher levels of CF, or completely the opposite, with younger ages indicating higher levels of CF [48,49]. As for CS, the data are very conflicting. As in this research, some studies have determined that age influences CS, although in a different way to the findings of the present study, instead finding that older ages indicated higher levels of CS [26,48]. In other cases, such as the study by Yu et al. [28] on oncology nurses, no relationship was found between these two variables. This diversity of results may perhaps be due to these studies having been carried out with nursing professionals who worked in very specific services, such as oncology services, acute admissions units, critical care units, and emergency departments, among others. This research therefore aims to provide more conclusive and decisive results, since it is based on a sample of nursing professionals who work in different settings and work environments.

With respect to the sex variable, differences in CS were significant, but not in the rest of the quality of life sub-dimensions. CS was significantly higher in women than in men [24]. Conversely, Mooney et al. [50] found that men had significantly higher CS levels than women. In turn, Hunsaker et al. [48] did not find any difference between men and women. The level of education and the cultural context may influence health professionals’ perceptions of responsibility and duty towards caring in different work environments, as with other care systems [41]. These relationships may be the result of the socio-cultural environment in which the research is conducted, since, from a cultural perspective, the task of caregiving in both the formal and informal spheres is conceived and developed by women. This might lead to a greater predisposition to develop compassionate empathy skills and to cultivate compassion, which is a protective factor against CF and a key element in attaining higher levels of CS.

Regarding the marital status of nursing professionals, the results of this study show that being married is a predictor of having a higher CF, while being divorced is a predictor of a higher CS. However, the studies by Yu et al. and Gómez-Martínez et al. [28,45] did not report statistically significant values for these variables. Haik et al. [51] found that being divorced was statistically related to having higher CF scores, although this study analysed medical personnel only. The marital status variable is related to the perception of social support. Perhaps, in the work context, the perceived social support of having a stable partner is not a factor influencing CF or CS. Nursing professionals seek support more from co-workers [52] or spiritual beliefs [53] than from personal relationships.

The healthcare setting has been found to be a factor influencing the professionals’ quality of life. CF was higher in primary care professionals, with primary care being a predictor of CF. In contrast, CS was higher among professionals working in hospitals [54]. Perhaps this relationship is the result of the close emotional bond that may be established with patients and their families throughout the entire life cycle in the community setting [7]. In this setting, bonds may become more sustained and intimate, and the strain of seeing individuals in their own natural environment in times of hardship may cause greater CF. In addition, there is a shortage of resources for good-quality home-based care, as well as a lack of replacements for patient and family care. In hospitals, professionals, if needed, may request the rotation of the care provided and, in most cases, encounters are brief and admission times are short [41,54]. Promoting self-compassion and supporting the delivery of compassionate care within the primary healthcare team may improve the care experienced by patients as well as the positive engagement and satisfaction of healthcare professionals. Compassion literacy enables practice nurses to provide compassionate care to their patients and identify factors that may limit it [55]. The specific work shift, and the morning/evening/night shift in particular, is a factor that influences CF, CS, and BO. This finding is important, since rotating shifts are a fundamental element of the continuity of care in nursing practice. Further studies are needed to delve into this organisational aspect, which may be key to improving quality of life among professionals. However, this factor has not been taken into account in other studies, unlike years of experience [27].

The present research has several limitations. First, a cross-sectional design was used. A longitudinal study would have been necessary to ascertain that the variables analysed have a cause–effect relationship. Secondly, social desirability bias may be present in the completion of the questionnaires. Thirdly, other variables, such as level of education or training in emotional coping skills for dealing with situations of suffering, have not been analysed [24]. Finally, there is a high proportion of women in the sample, a characteristic feature of the nursing profession, who also work in urban areas. This is only natural, as the majority of the healthcare centres are located in cities.

Despite these limitations, this research relies on a wide and varied sample that may provide relevant information on the socio-demographic and work-related variables concerning the quality of life of nursing professionals.

## 5. Conclusions

The data obtained in our study show high levels of CF and BO, and below-average CS levels in nursing professionals. All of this affects their quality of life. Socio-demographic factors such as marital status, the healthcare setting, the area where the workplace is located, and the work shift are directly related to CF. In contrast, the following variables are involved in the CS sub-dimension: age, sex, marital status, the healthcare setting, the area where the workplace is located, and the work shift. In turn, the work shift is related to the occurrence of BO.

Future lines of research emerge on the basis of these data. Firstly, future studies should determine which are the healthcare services that most affect or disrupt the well-being of nursing professionals and delve into the factors that affect their professional quality of life. Subsequently, employment and organisational measures should be implemented accordingly, and specific interventions should be designed based on the characteristics of the population being cared for, as well as on the specific context of hardship in the lives of patients requiring the professionals’ support. These interventions should focus on cultivating compassion. Cultivating compassion empowers professionals with the ability to be present in contexts of suffering and the genuine desire to prevent and/or alleviate such suffering, thereby diminishing CF and increasing CS, which is a protective factor for professionals. The wellbeing of nursing professionals and the quality of patient care will thus be improved. Cultivating compassion must be a core element in future nursing intervention programmes.

## Figures and Tables

**Table 1:** Socio-demographic and work-related characteristics of the participants.

Variables	% (*n*)	Variables	% (*n*)
Sex		Location	
Female	75.5% (1148)	Urban area	89.5% (1361)
Male	24.5% (373)	Semi-urban area	5.5% (83)
Marital status		Rural area	5.1% (77)
Married	69.8% (1062)	Work shift	
Single	15.8% (241)	Mornings	32.1% (488)
Divorced	6.5% (99)	Evenings	0.9% (13)
Other	7.8% (119)	Mornings/Evenings	23.1% (351)
Employment status		Mornings/Evenings/Nights	29% (441)
Casual	13.3% (203)	12-h	13.5% (205)
Temporary or long-term	27.2% (413)	Nights	0.7% (10)
Statutory or permanent	59.5% (905)	Other	0.9% (13)
Healthcare setting		Variables	M (SD)
Hospital care	55% (836)	Work experience (months)	275.36 (110.42)
Primary care	45% (685)	Seniority in the current position (months)	146.81 (117.52)

*n* = number of participants; % = percentages; M = mean; SD = standard deviation.

**Table 2:** Socio-demographic variables, work-related variables, and quality of life.

Variables	CF		CS		BO	
	M (SD)	Values	M (SD)	Values	M (SD)	Values
Age	20.74 (7.88)	r = 0.02 *p* = 0.36	35.48 (7.39)	r = −0.61 *p* = 0.01 *	23.44 (5.29)	r = 0.01 *p* = 0.64
Sex						
Female	20.74 (0.23)	t = −0.02 *p* = 0.98	35.7 (0.21)	t = −0.01 *p* = 0.04 *	23.40 (0.15)	t = −0.01 *p* = 0.79
Male	20.75 (0.42)		34.82 (0.38)		23.50 (0.29)	
Marital status						
Married	21.10 (7.71)	F = 3.33 *p* = 0.01 *	35.38 (7.19)	F = 2.95 *p* = 0.03 *	23.58 (5.17)	F = 1.38 *p* = 0.24
Single	20.16 (7.89)		35 (8.07)		23.29 (5.45)	
Divorced	18.77 (8.92)		37.51 (7.65)		22.49 (5.84)	
Other	20.37 (8.16)		37.75 (7.36)		23.9 (5.47)	
Employment status						
Casual	20.51 (8.40)	F = 0.55 *p* = 0.57	36.36 (7.66)	F = 2.14 *p* = 0.11	23.14 (5.53)	F = 0.37 *p* = 0.68
Temporary or long-term	20.47 (7.96)		35.65 (7.38)		23.50 (5.48)	
Statutory or permanent	20.91 (7.72)		35.21 (7.33)		23.48 (5.14)	
Healthcare setting						
Hospital care	20.37 (7.66)	t = −2.03 *p* = 0.04 *	35.78 (7.39)	t = −1.75 *p* = 0.07	23.50 (5.19)	t = 0.51 *p* = 0.61
Primary care	21.19 (7.99)		35.12 (7.39)		23.36 (5.40)	
Location						
Urban area	20.92 (7.88)	F = 3.31 *p* = 0.03 *	35.17 (7.40)	F = 11.83 *p* = 0.01 *	23.51 (5.27)	F = 1.18 *p* = 0.30
Semi-urban area	9.35 (7.93)		37.98 (6.95)		22.98 (5.47)	
Rural area	19.10 (7.51)		38.34 (6.6)		22.70 (5.37)	
Work shift						
Mornings	20.13 (7.80)	F = 3.45 *p* = 0.00 *	36.28 (7.31)	F = 2.52 *p* = 0.01 *	22.94 (5.22)	F = 2.44 *p* = 0.02
Evenings	22.46 (8.26)		34.54 (7.10)		24.54 (6.30)	
Mornings/Evenings	21.63 (8.17)		34.74 (7.43)		23.58 (5.56)	
Mornings/Evenings/Nights	21.38 (7.59)		34.83 (7.29)		23.98 (5.01)	
12-h shift	19.60 (7.95)		36.23 (7.36)		23.42 (5.40)	
Nights	16.70 (7.15)		36.40 (8.42)		20.90 (5.78)	
Other	17.23 (6.67)		36.08 (10.07)		21.15 (4.72)	
Work experience (months)	275.36 (110.42)	r = 0.01 *p* = 0.82	275.36 (110.42)	r = −0.02 *p* = 0.27	275.36 (110.42)	r = −0.01 *p* = 0.65
Seniority in the current position (months)	146.81 (117.52)	r = 0.01 *p* = 0.52	146.81 (117.52)	r = −0.02 *p* = 0.41	146.81 (117.52)	r = 0.16 *p* = 0.55

CF: compassion fatigue; CS: compassion satisfaction; BO: burnout; M = mean; SD = standard deviation; *p* = level of statistical significance; t = Student’s *t*-test; F = one-way ANOVA; r = Pearson correlation; * Correlation is significant at the 0.05 level.

**Table 3:** Linear regression model: socio-demographic variables, work-related variables, and CF.

**Models**	**R**	**R** **^2^**	**Adjusted R^2^**	**Standard Error of the Estimate**	**Change Statistics**	
					**R** **^2^** **Change**	**F** **Change**
1	0.06 ^a^	0	0	7.86	0	7.26
2	0.09 ^b^	0	0	7.85	0	6.68
3	0.12 ^c^	0.01	0.01	7.83	0	8.92
4	0.14 ^d^	0.02	0.02	7.9	0	9.06
**Model 4**	**Unstandardised Coefficients**		**Standardised Coefficients**			**95% Confidence Interval**
	**B**	**Std. Error**	**Beta**	**t**	***p***	**Lower Limit**
(Constant)	16.6	0.81		20.3	0	15
Married	1.17	0.43	0.06	2.68	0	0.31
Urban area	2.36	0.69	0.09	3.42	0	1.01
Primary care	1.74	0.45	0.11	3.84	0	0.85
Morning/Evening/Night shift	1.42	0.47	0.08	3.01	0	0.49

Note: R = coefficient of determination; F = Fisher-Snedecor test; β = Regression coefficient; Std. Error= Standard Error; t = Student’s *t*-test; a = married; b = married, urban area; c = married, urban area, primary care; d = married, urban area, primary care, morning/evening/night shift. Correlation is significant at the 0.05 level.

**Table 4:** Regression model: socio-demographic variables, work-related variables, and CS.

**Models**	**R**	**R** **^2^**	**Adjusted** **R** **^2^**	**Standard Error of the Estimate**	**Change Statistics**		
					**R** **^2^** **Change**	**F** **Change**	**Sig.** **F** **Change**
1	0.126 ^a^	0.01	0.01	7.34	0.01	24	0
2	0.153 ^b^	0.02	0.02	7.31	0	11.51	0
3	0.169 ^c^	0.02	0.02	7.29	0	8.29	0
4	0.182 ^d^	0.03	0.03	7.28	0	6.92	0
5	0.191 ^e^	0.04	0.04	7.27	0	5.52	0.01
**Model 5**	**Unstandardised Coefficients**		**Standardised Coefficients**			**95% Confidence Interval**	
	**B**	**Std. Error**	**Beta**	**t**	***p***	**Lower Limit**	**Upper Limit**
(Constant)	42.23	1.26		33.49	0	39.75	44.7
Urban area	−3.59	0.65	−0.14	−5.52	0	−4.87	−2.31
Primary care	−1.63	0.43	−0.11	−3.8	0	−2.48	−0.79
Morning/Evening/Night shift	−1.39	0.44	−0.08	−3.11	0	−2.26	−0.51
Divorced	2.16	0.77	0.07	2.8	0	0.65	3.67
Age	−0.05	0.02	−0.06	−2.35	0.01	−0.1	0

Note: R = coefficient of determination; F = Fisher-Snedecor test; β = Regression coefficient; t = Student’s *t*-test; a = urban area; b = urban area, primary care; c = urban area, primary care, morning/evening/night shift; d = urban area, primary care, morning/evening/night shift, divorced; e = urban area, primary care, morning/evening/night shift, divorced, age. Correlation is significant at the 0.05 level.

**Table 5:** Regression model: socio-demographic and work-related variables, and BO.

**Model**	**R**	**R^2^**	**Adjusted R^2^**	**Standard Error of the Estimate**	**Change Statistics**		
					** R ** **^2^** **Change**	**F Change**	**Sig. *F* Change**
1	0.06	0.04	0.04	5.28	0.04	6.58	0.01
**Model 5**	**Unstandardised Coefficients**		**Standardised Coefficients**			**95% Confidence Interval**	
	***B***	**Std. Error**	**Beta**	***t***	***p***	**Lower Limit**	**Upper Limit**
(Constant)	23.21	0.16		144.44	0.00	22.9	23.53
Morning/Evening/Night shift	0.76	0.29	0.66	2.56	0.01	0.18	1.35

Note: R = coefficient of determination; F = Fisher-Snedecor test; β = Regression coefficient; t = Student’s *t*-test. Correlation is significant at the 0.05 level.
